# High mobility group protein A2 overexpression indicates poor prognosis for cancer patients: a meta-analysis

**DOI:** 10.18632/oncotarget.23085

**Published:** 2017-12-10

**Authors:** Dan Nie, Lingping Zhang, Qian Guo, Xiguang Mao

**Affiliations:** ^1^ Department of Gynecology, The Affiliated Hospital of Southwest Medical University, Luzhou, Sichuan, People’s Republic of China; ^2^ Department of Neonatology, The Affiliated Hospital of Southwest Medical University, Luzhou, Sichuan, People’s Republic of China

**Keywords:** high-mobility group protein A, prognosis, meta-analysis

## Abstract

Overexpression of the high mobility group protein A2 (HMGA2), an architectural transcription factor, has been linked to poor prognosis in many malignancies, although this remains controversial. Herein, we conducted a meta-analysis to investigate whether HMGA2 has prognostic value, and evaluated the association between HMGA2 and clinicopathologic factors in malignancies. A total of 29 studies involving 4114 patients were included in this meta-analysis. The pooled results demonstrated that elevated HMGA2 predicted a poor overall survival (OS) (hazard ratio [HR] = 1.82; 95% confidence interval [CI] = 1.62–2.05; *P* < 0.001) and disease-free survival/progression-free survival/recurrence-free survival (HR = 1.94; 95% CI = 1.27–2.98; *P* = 0.002). Subgroup analysis conducted by study region, sample size, detection method, and analysis method indicated that HMGA2 overexpression correlated with poor OS. Furthermore, HMGA2 overexpression was found to be linked to poor OS in various cancers except ovarian cancer (pooled HR = 1.14; 95% CI = 0.62–2.09; *P* = 0.673). High HMGA2 expression level also correlated with advanced TNM stage (OR = 2.44; 95% CI =1.87–3.2; *P* < 0.001), lymphovascular invasion (OR = 2.46, 95% CI = 1.67–3.64; *P* < 0.001), distant metastasis (OR = 2.66; 95% CI =1.51–4.69; *P* < 0.001), and lymph node metastasis (OR = 1.83; 95% CI =1.27–2.64; *P* = 0.001). In conclusion, HMGA2 overexpression indicates a worse prognosis and may serve as a prognostic predictor in cancer patients.

## INTRODUCTION

Cancer is a major public health problem worldwide and the leading cause of death in China [1]. Based on the GLOBOCAN estimates of cancer morbidity and mortality, approximately 14.1 million new cases and 8.2 million deaths occurred in 2012 [2]. The number of cancer survivors has increased steadily because of the advances in early detection and treatment as well as the aging and growth of the population [3]. However, the 5-year survival rate of most cancers is still low, many patients are asymptomatic and often diagnosed in an advanced disease stage [4]. Therefore, it is necessary to identify new prognostic and diagnostic markers that can help clinicians identify patients with a poor prognosis and use more efficient treatment strategies.

High-mobility group AT-hook 2 (HMGA2), a member of the high mobility group (HMG) protein family, is a non-histone chromosomal protein [5, 6]. It modulates gene transcription by interacting with various transcription factors and altering the chromatin structure [6, 7], and is a known regulator of cell growth, differentiation, apoptosis, and DNA repair [8, 9]. High expression of HMGA2 has been detected in both epithelial and mesenchymal tissue of malignant tumors, and may promote tumorigenesis [9]. Recent studies have revealed that the overexpression of HMGA2 correlates with higher lymph node metastasis rates, poor tumor differentiation, and unfavorable prognosis [10–14], implying that HMGA2 has prognostic value in cancer.

Recently, a meta-analysis in gastric cancer indicated that HMGA2 overexpression is associated with poor prognosis of cancer patients [15]. However, only six studies involving 712 gastric cancer patients were analyzed in this study. Moreover, whether overexpression of HMGA2 has prognostic value in other cancer types has not been evaluated. Therefore, we conducted a meta-analysis of relevant studies to understand if HMGA2 has predictive prognostic potential in various cancers.

## RESULTS

### Characteristics of eligible studies

**Figure 1:**
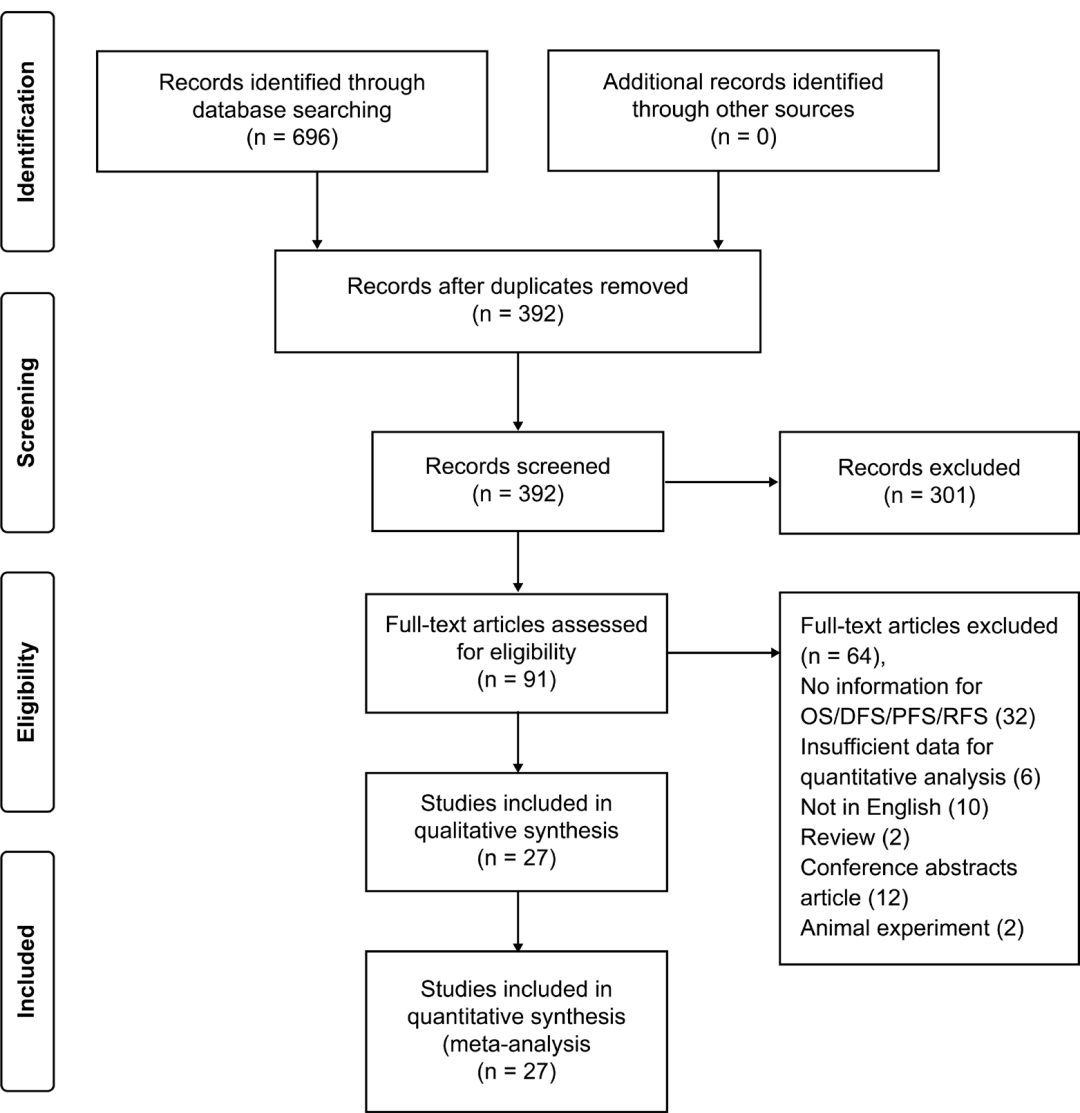
Flow chart of study selection strategy

A total of 696 articles were identified after the primary search, of which 304 duplicate articles were removed, and a further 301 articles were excluded after reviewing the title and abstract. The full texts of the remaining 91 articles were reviewed, and additional exclusions were made: 32 studies: survival data not reported; 6 studies: insufficient data for quantitative analysis, 10 studies: not written in English; 14 studies: conference abstracts or reviews; and 2 studies: involved animal experiments. Finally, 27 qualifying studies were enrolled for this meta-analysis (Figure 1).

Among the 27 articles, two studies included two different cohorts with survival data, so that 29 studies including a total number of 4114 patients were analyzed. These studies were mainly conducted in East Asia, Europe, and the United States of America. The cancer types studied were: gastric cancer (GC) [16–18], breast cancer (BC) [10, 19], hepatocellular carcinoma (HCC) [11, 13], colorectal cancer (CRC) [14, 20, 21], ovarian cancer (OC) [22, 23], nasopharyngeal carcinoma (NPC) [24, 25], esophageal carcinoma (EC) [26, 27], head and neck squamous cell carcinoma (HNSCC) [28–30], clear cell renal cell carcinoma [12], intrahepatic cholangiocarcinoma [31], glioblastoma [32], gallbladder adenocarcinoma [33], melanoma [34], non-small-cell lung cancer [35], and bladder cancer [36]. HMGA2 was detected using immunohistochemistry (IHC) (24 studies), reverse transcription-polymerase chain reaction (RT-PCR) (2 studies), or quantitative real-time polymerase chain reaction (qRT-PCR) (3 studies). The expression of HMGA2 was correlated with overall survival (OS) in 25 studies, and disease-free survival (DFS)/progression-free survival (PFS)/recurrence-free survival (RFS) in 4 studies. Studies were assessed using the Newcastle-Ottawa Scale (NOS). Supplementary Table 1 displays the general characteristics of the qualifying studies.

### Correlation between HMGA2 expression and OS

**Figure 2:**
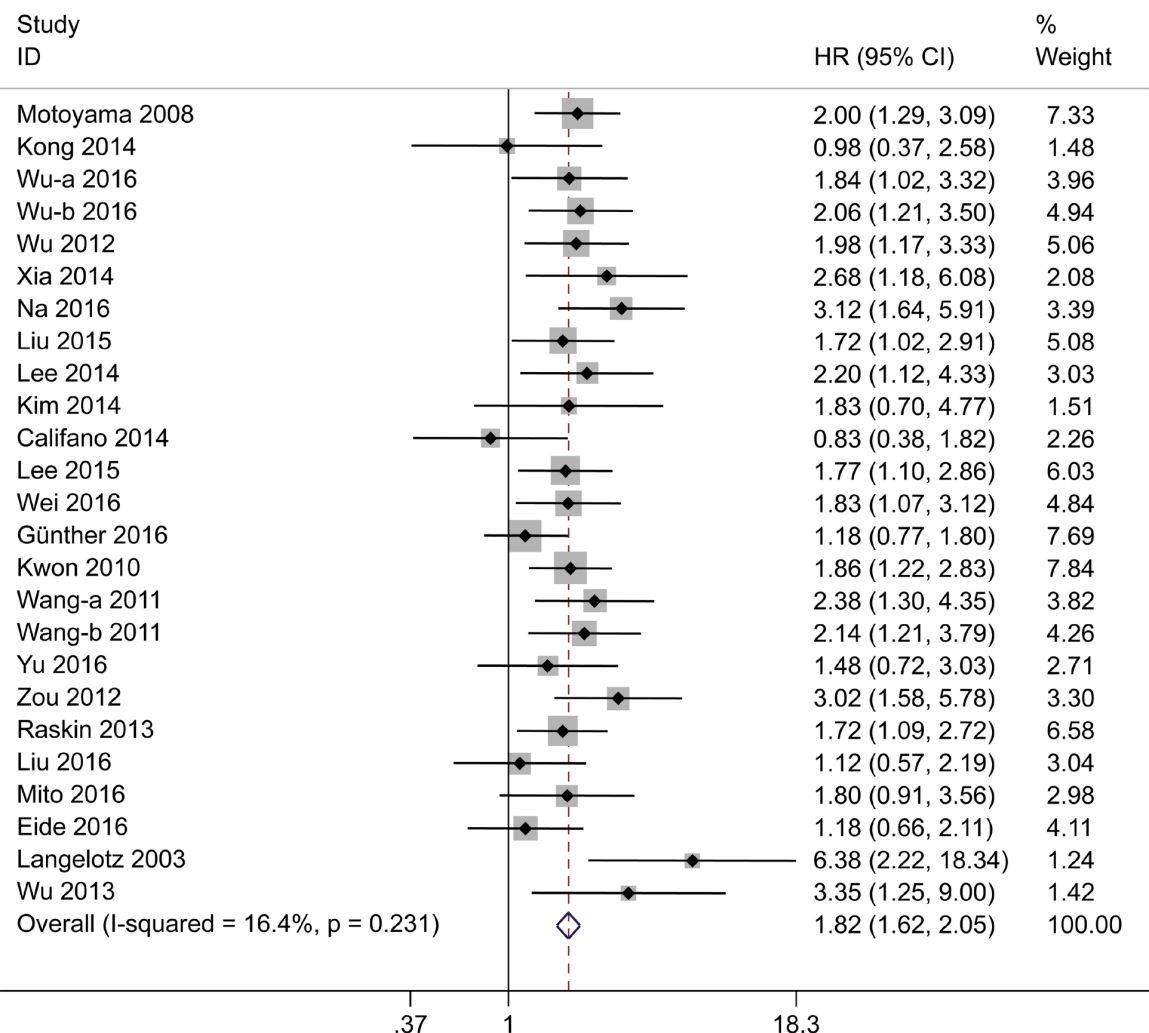
Forest plot to assess the association of HMGA2 expression with overall survival

Combined analysis of 25 studies suggested that the overexpression of HMGA2 correlated with poor OS of cancer patients (pooled hazard ratio [HR] = 1.82; 95% confidence interval [CI] = 1.62–2.05; *P* < 0.001). Low heterogeneity was observed between these studies (*I*^2^ = 16.4%; *P* = 0.231; fixed effects) (Figure 2). Subgroup analysis was conducted based on study region, sample size, detection method, analysis method, and cancer type (Table 1). Subgroup analysis by cancer type showed that high HMGA2 expression was associated with worse OS in GC (pooled HR = 1.77; 95% CI =1.31–2.41; *P* < 0.001), BC (pooled HR = 2.26; 95% CI =1.56–3.28; *P* < 0.001), HCC (pooled HR = 1.90; 95% CI =1.37–2.64; *P* < 0.001), CRC (pooled HR = 1.78; 95% CI =1.29–2.44; *P* < 0.001), NPC (pooled HR = 1.96; 95% CI =1.26–3.05; *P* = 0.003), and EC (pooled HR = 1.82; 95% CI =1.19–2.77; *P* = 0.006). However, in ovarian cancer (pooled HR = 1.14; 95% CI = 0.62–2.09; *P* = 0.673), HMGA2 overexpression did not prognosticate OS. In subgroup analysis by study region, patients from both Asian (HR = 1.95; 95% CI = 1.69–2.25; *P* < 0.001) and non-Asian regions (HR = 1.60; 95% CI = 1.14–2.25; *P* = 0.007) showed a significant correlation between high HMGA2 expression and poor OS. Pooled HR results for subgroup analyses by sample size, detection method and analysis method were >1 in all subgroups.

**Table 1:** Correlation between HMGA2 expression and overall survival in cancer patients: subgroup analyses

Categories	Studies	Number of patients	Model	HR (95% CI)	*I*^*2*^ (%)	*P*_*h*_	*Z*	*P*
Cancer type								
GC	3	438	Fixed	1.77 (1.31–2.41)	0.0%	0.418	3.67	<0.001
BC	3	652	Fixed	2.26 (1.56–3.28)	53.4%	0.117	4.33	<0.001
HCC	2	441	Fixed	1.90 (1.37–2.64)	0.0%	0.861	3.85	<0.001
CRC	4	547	Fixed	1.78 (1.29–2.44)	11.5%	0.335	3.56	<0.001
OC	2	187	Fixed	1.14 (0.62–2.09)	36.3%	0.210	0.42	0.673
NPC	2	240	Fixed	1.96 (1.26–3.05)	0.0%	0.372	3.00	0.003
EC	2	187	Fixed	1.82 (1.19–2.77)	0.0%	0.975	2.78	0.006
Other	7	1062	Random	1.87 (1.35–2.60)	52.4%	0.05	3.77	<0.001
Study region								
Asian	17	2549	Fixed	1.95 (1.69–2.25)	0.0%	0.764	9.16	<0.001
Non-Asian	8	1205	Random	1.60 (1.14–2.25)	51.3%	0.045	3.18	0.001
Sample size								
≥100	17	3156	Fixed	1.75 (1.53–2.00)	20.3%	0.217	8.23	<0.001
<100	8	598	Fixed	2.14 (1.66–2.75)	0.0%	0.457	5.87	<0.001
Detection method								
IHC	21	3085	Fixed	1.81 (1.59–2.07)	4.6%	0.400	8.81	<0.001
RT-PCR	2	403	Fixed	1.69 (1.19–2.18)	0.0%	0.591	6.66	<0.001
qRT-PCR	2	266	Fixed	1.66 (1.17–2.34)	50.7%	0.154	2.84	0.005
Analysis method								
Multivariate	20	3145	Fixed	1.91 (1.68–2.18)	14.3%	0.276	9.72	<0.001
Univariate	5	609	Fixed	1.49 (1.14–1.96)	0.0%	0.408	2.89	0.004

Notes: Random-effects model was used when *P*-value for heterogeneity test <0.1; otherwise, fixed-effects model was used.

Abbreviations: GC: gastric cancer; BC: breast cancer; HCC: hepatocellular carcinoma; CRC: colorectal cancer; OC: ovarian cancer; NPC: nasopharyngeal carcinoma; EC: esophageal carcinoma; *P*_*h*_*: P*-value for heterogeneity based on *Q*-test; *P: P*-value for statistical significance based on *Z*-test; HR: hazard ratio; IHC: immunohistochemistry; RT-PCR: reverse transcription-polymerase chain reaction; qRT-PCR: quantitative real-time polymerase chain reaction.

### Correlation between HMGA2 expression and clinicopathological features

**Figure 3:**
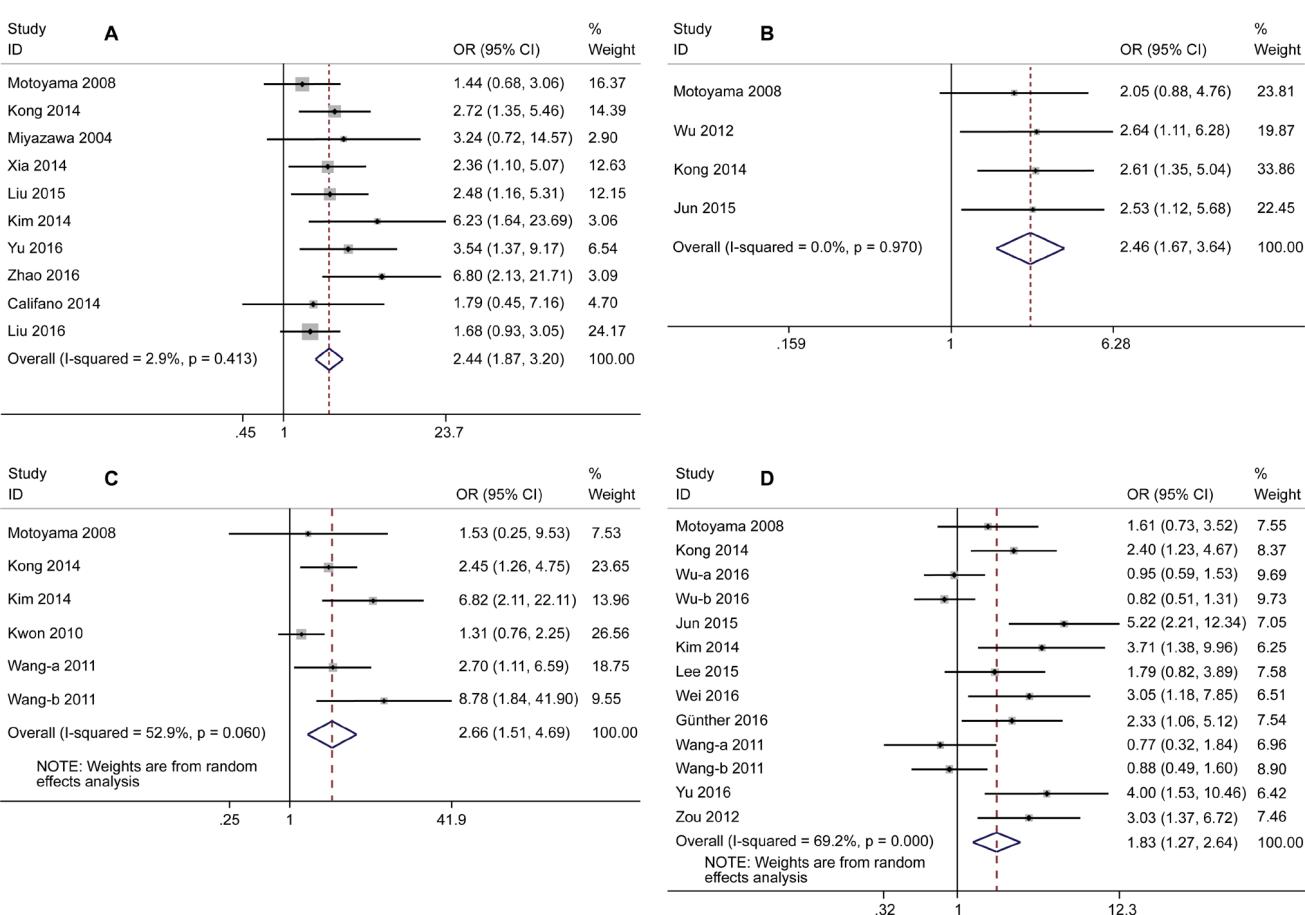
Forest plots of correlation between odds ratio and TNM stage (I/II *vs.* III/IV) (**A**), lymphovascular space invasion (negative *vs.* positive) (**B**), distant metastasis (absent *vs.* present) (**C**), lymph node metastasis (negative *vs.* positive) (**D**).

The correlation between HMGA2 expression and clinicopathologic features is shown in Figure 3A–3D. High HMGA2 expression was related to advanced tumor node metastasis (TNM) stage (stage III/IV) (odds ratio [OR] = 2.44; 95% CI =1.87–3.2; *P* < 0.001), positive lymphovascular space invasion (OR = 2.46, 95% CI = 1.67–3.64; *P* < 0.001), distant metastasis (OR = 2.66; 95% CI = 1.51–4.69; *P <* 0.001), and lymph node metastasis (OR = 1.83; 95% CI = 1.27–2.64; *P* = 0.001).

### Correlation between HMGA2 expression and DFS/PFS/RFS

**Figure 4:**
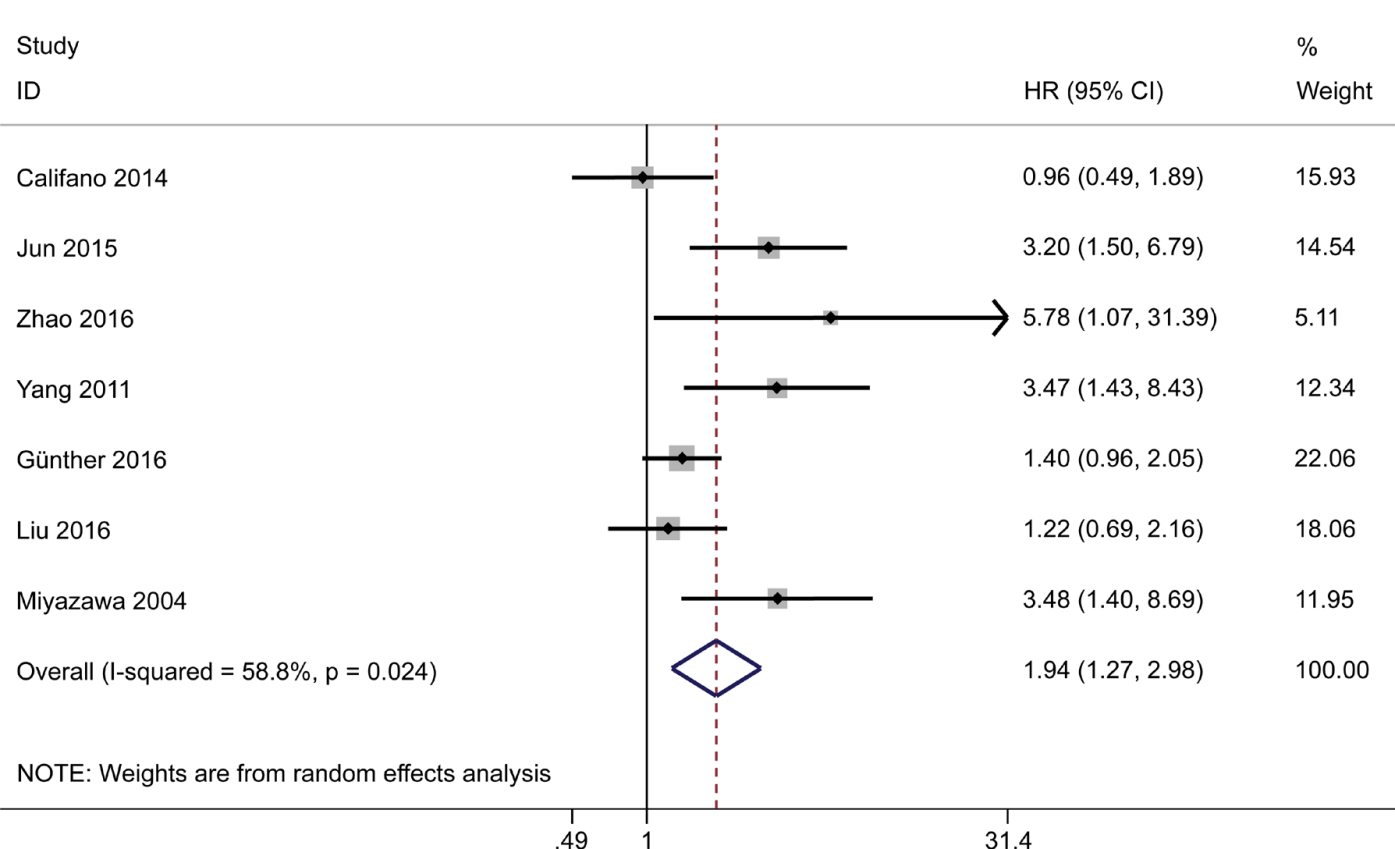
Forest plot to assess the association of HMGA2 expression with disease-free survival/progression-free survival/ recurrence-free survival

Analysis of data pooled from seven studies that reported DFS/PFS/RFS (three of which included both OS and DFS/PFS/RFS data) showed that overexpression of HMGA2 predicted a poor DFS/PFS/RFS in the random effects model (HR = 1.94; 95% CI = 1.27–2.98; *P* = 0.002). A high degree of heterogeneity was detected among the studies (*I*^*2*^ = 58.8%, *P* = 0.024) (Figure 4). Subgroup analysis was not performed due to the small number of studies in this category.

### Publication bias and sensitivity analysis

**Figure 5:**
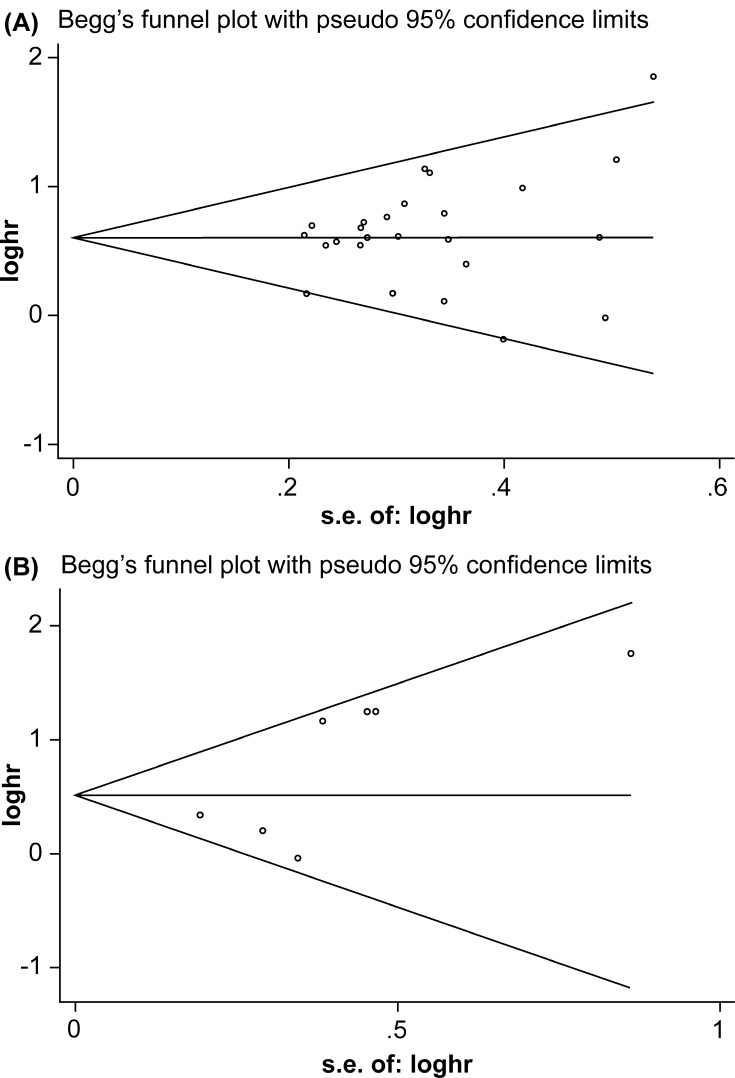
(**A**) Funnel plot for the meta-analysis of correlation between HMGA2 expression and overall survival. (**B**) Funnel plot for the meta-analysis of the correlation between HMGA2 expression and disease-free survival/progression-free survival/ recurrence-free survival.

**Figure 6:**
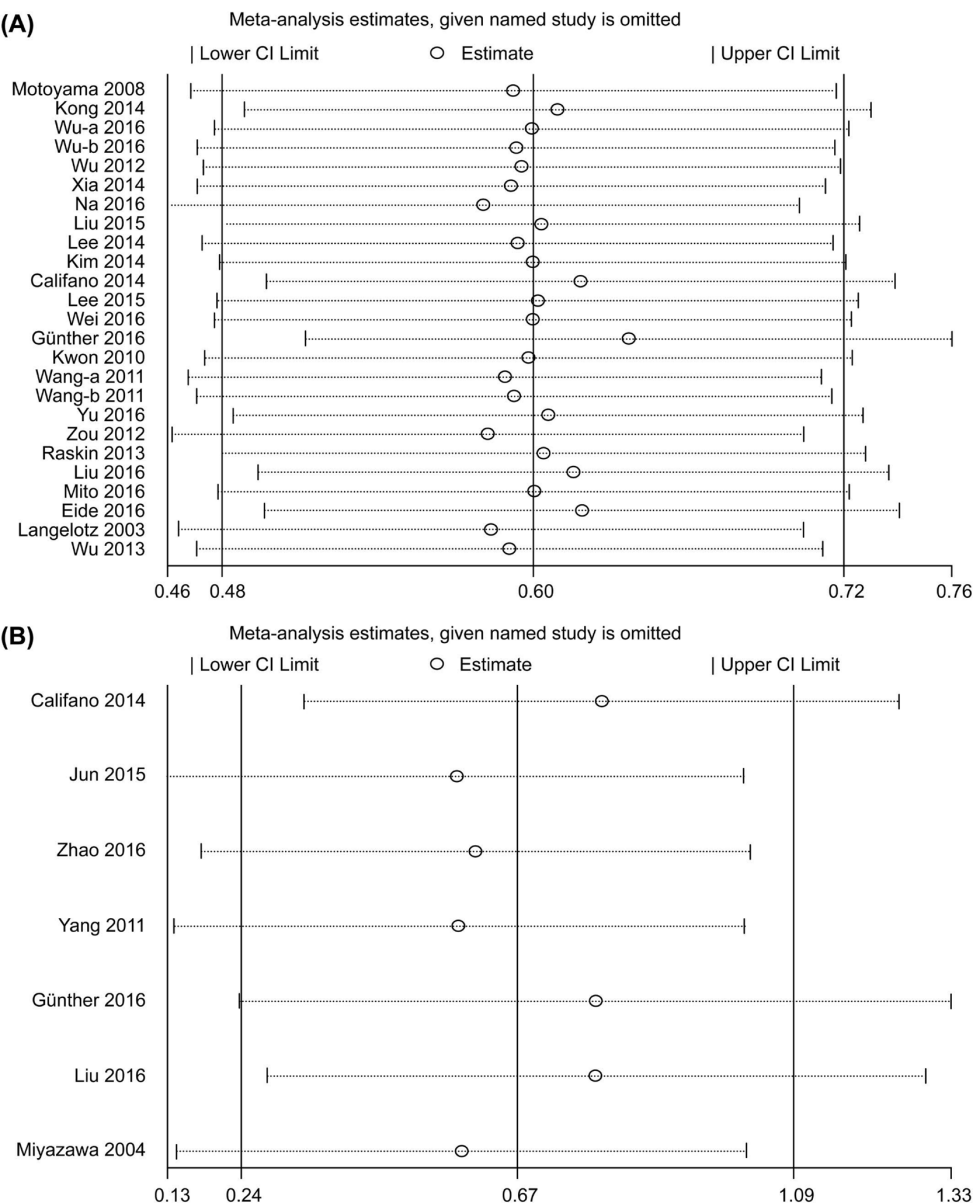
(**A**) Sensitivity analysis of the association between HMGA2 expression and overall survival. (**B**) Sensitivity analysis of the association between HMGA2 expression and disease-free survival/progression-free survival/recurrence-free survival.

Begg’s test and Egger’s test were applied to assess publication bias. The results indicated that there was no obvious publication bias for OS (*P* = 0.199 for Begg’s test and 0.271 for Egger’s test) or DFS/PFS/RFS (*P* = 0.764 for Begg’s test and *P* = 0.076 for Egger’s test) among the included studies (Figure 5A and 5B). To ensure stability of results, we performed sensitivity analysis by sequentially omitting each study and analyzing the remaining datasets. We found that none of the studies had a significant effect on the OS or DFS/PFS/RFS (Figure 6A and 6B) independent of the others. This confirmed that the outcomes were stable and reliable.

## DISCUSSION

The HMGA protein family contains HMGA1a, HMGA1b, HMGA1c, and HMGA2 proteins [37]. Recently, evidence has implicated HMGA2 having complex functions in cancer [9]. HMGA2 promotes tumorigenesis through regulating transcription of human telomerase reverse transcriptase (hTERT) and binding directly to the promoters of FN1 and IL11 [38, 39]. Moreover, aberrant expression of HMGA2 promotes cancer invasion, metastasis, and epithelial-to-mesenchymal transition (EMT) by activating the transforming growth factor beta (TGFβ) and Wnt/β-catenin signaling pathways [40, 41]. In addition, HMGA2 through activating HMGA2-FOXL2-ITGA2 and HMGA2-TET1-HOXA9 pathways prompts cancer distant metastasis and chemoresistance [42, 43]. Furthermore, miRNAs like miR-490-3p and miR-145 can inhibit cancer development and progression by direct regulation of HMGA2 expression [23, 44]. A study by Li *et al.* [45] showed that long non-coding ribonucleic acid (lncRNA) HIT000218960 promotes papillary thyroid cancer by upregulating HMGA2 expression. These results suggest that HMGA2 acts as a target gene of many micro RNAs (miRNAs) and lncRNAs, which play critical roles in regulating tumorigenesis and cancer progression. In addition, several recent studies demonstrated that HMGA2 overexpression, both in the tissues and blood of patients with cancer correlated with poor tumor differentiation, positive lymph node metastasis, and advanced stage, indicating a poor prognosis [46, 47]. As a member of the HMGA protein family, HMGA1 also has been reported to be overexpressed in cancers and promote tumorigenesis [48–51]. However, the prognostic role of HMGA1 in different types of cancers remains controversial. Liau *et al.* [52] suggested that HMGA1 overexpression promotes tumorigenicity through activating PI3-K/Akt-dependent signaling pathways and predicts poor postoperative survival of pancreatic adenocarcinoma patients. While Jun *et al.* [18] indicated that HMGA1 overexpression was not correlated with lymphatic invasion, TNM stage, or cancer recurrence. Therefore, further studies are needed to clarify the prognostic role of HMGA1 in different types of cancers. In this meta-analysis, we only sought to understand whether HMGA2 can be used as a prognostic biomarker in cancer patients.

In this meta-analysis, we included 4114 patients from 29 studies, and the outcomes demonstrated a statistically significant correlation between high HMGA2 expression and poor OS. Meanwhile, high HMGA2 expression was significantly correlated with short DFS/PFS/RFS. In subgroup analysis, we found that high expression of HMGA2 conferred a worse OS in patients regardless of the study region, sample size, detection method, or analysis method, which further confirmed the prognostic potential of HMGA2. Subgroup analysis by cancer type revealed a significant correlation between HMGA2 expression and OS in gastric cancer, breast cancer, hepatocellular carcinoma, colorectal cancer, nasopharyngeal carcinoma, and esophageal carcinoma. However, no such correlation was seen in ovarian cancer. A study by Hetland *et al.* [53] has suggested that HMGA2 expression was unrelated to chemotherapy response or survival of ovarian serous carcinoma patients. Meanwhile, according to Califano *et al.* [22], overexpression of HMGA2 has no significant prognostic value for DFS and OS in multivariate analysis; even high HMGA2 expression combined with high body mass index (BMI; ≥25 kg/m^2^) indicated a poor prognosis in patients with ovarian cancer. Our outcome is consistent with results of these studies. However, there were only 2 studies included in this subgroup, so publication bias may have contributed to the negative outcome. Lymph node metastasis, lymphovascular space involvement, and advanced stage significantly affect survival outcomes of cancer patients [54]. According to our results, HMGA2 overexpression was significantly linked to advanced TNM stage (stage III/IV), positive lymphovascular space invasion, distant metastasis, and lymph node metastasis. This further verifies the prognostic role of HMGA2.

There are several limitations to this meta-analysis. First, only studies published in English were included. Since authors are more likely to publish positive results in an English-language journal [55], negative outcomes published in non-English journals may have been omitted from the analysis, leading to language bias. Second, differences in detection methods, interpretation of results, and parameter cut-off values among the included studies may have confounded the analyzed outcomes. In our included 29 studies, 13 studies defined the overexpression and low expression of HMGA2 by multiplying the scores of expression intensity and positivity area. Seven studies defined the overexpression and low expression by percentage of positive staining cells. Only one study defined the overexpression and low expression by the median HMGA2 mRNA expression level. Additionally, the rest eight studies did not report the cutoff values. All these studies reported that HMGA2 may be a promising prognostic factor for cancer patients. However, more studies are needed to reach a consensus for different methods and criteria for overexpression of HMGA2. Third, some studies did not provide OS or DFS/PFS/RFS data directly, which were subsequently extracted from Kaplan–Meier curves in such cases. This may have reduced the credibility of the final results. Fourth, subgroup analysis could not eliminate heterogeneity across studies completely, which may have led to biases. Moreover, all the selected studies are small sample retrospective studies, which may cause reporting bias. Therefore, further larger, multi-center, high-quality prospective investigations are required to overcome the above-mentioned limitations.

## CONCLUSIONS

In conclusion, this meta-analysis confirmed that high HMGA2 expression in cancer is linked to poor prognosis, and HMGA2 is a potential predictive biomarker for OS. Additional larger, well-designed multicenter prospective studies are necessary to confirm our results.

## MATERIALS AND METHODS

### Search strategy

A comprehensive search was performed in PubMed, EMBASE, and Web of Science databases to identify relevant articles published from 1996 to December 31, 2016. The search terms were as follows: (“HMGA2 Protein”, or “High-mobility group A2”, or “HMGA2”), and (“neoplasm”, or “cancer”, or “carcinoma” or “tumor”), and (“prognosis”, or “prognostic” or “outcome”, or “survival’’). We also retrieved relevant systematic reviews and references to find additional eligible studies. This meta-analysis complies with the guidelines of the Preferred Reporting Items for Systematic Reviews and Meta-Analyses (Supplementary Table 2) [56].

### Selection criteria

Studies fitting the following inclusion criteria were analyzed: (1) correlation between the expression of HMGA2 and OS was assessed; (2) HMGA2 expression was analyzed by IHC, RT-PCR, or qRT-PCR; (3) patients were classified into high HMGA2 expression (or HMGA2-positive) and low HMGA2 expression (or HMGA2-negative) groups; (4) HR and 95% CI were provided or could be extracted indirectly; (5) the article was written in English; (6) when publications involved the same patient population, only the largest patient cohort was included. Studies were excluded as per the following criteria: (1) were reviews, letters, involved basic research, or were animal studies; (2) provided inadequate survival data.

The articles included in the study were evaluated by two investigators (DN and LPZ), independently. Any disagreement between the investigators was resolved by consensus.

### Data extraction and quality assessment

Two investigators (DN and LPZ) collected data from the selected articles independently. The author name(s), publication year, country, sample size, cancer type, clinic stage, HMGA2 detection method, follow-up time, and survival data including OS, DFS/PFS/RFS, were extracted. In studies including both univariate and multivariate analyses, only the HRs and the corresponding 95% CIs from multivariate analyses were considered. Otherwise, the HRs and CIs were either extracted from univariate analysis, or were calculated from the Kaplan-Meier curve using methods provided in literature [57]. Study quality was evaluated according to the NOS and a score of six points or higher signified high quality.

### Statistical analysis

The prognostic value of HMGA2 expression in predicting OS and PFS/RFS/DFS/ was evaluated by analyzing pooled HRs and respective 95% CIs. The pooled ORs and their 95% CIs were combined to assess the correlation between HMGA2 expression and TNM stage (I/II *vs.* III/IV), lymphovascular space invasion (negative *vs.* positive), distant metastasis (absent *vs.* present), and lymph node metastasis (negative *vs.* positive). Chi-square test based on the *Q* statistic and *I*^*2*^ statistic was used for heterogeneity analysis. When significant heterogeneity was observed (*P*-value < 0.1 and/or *I*^*2*^> 50%), random-effects model was used to analyze the pooled HRs. Otherwise, fixed-effects model was applied. Sensitivity analysis was conducted to validate the stability of the pooled outcomes. Publication bias was assessed using Begg’s test and Egger’s test. A *P*-value < 0.05 was defined as statistically significance. All statistical analyses were performed using STATA 14.0 software (Stata Corporation, College Station, TX, USA).

## SUPPLEMENTARY MATERIALS TABLES







## Abbreviations

BC: breast cancer
BMI: body mass index
CRC: colorectal cancer
DFS: disease-free survival
EC: esophageal carcinoma
EMT: epithelial-to-mesenchymal transition
GS: gastric cancer
HCC: hepatocellular
HMGA2: high mobility protein A2
HNSCC: head and neck squamous cell carcinoma
HR: hazard ratio
hTERT: human telomerase reverse transcriptase
IHC: immunohistochemistry
IncRNA: long non-coding ribonucleic acid
miRNAs: micro ribonucleic acids
NPC: nasopharyngeal carcinoma
OC: ovarian cancer
OR: odds ratio
OS: overall survival
PFS: progression-free survival
RFS: recurrence-free survival (RFS)
RT-PCR: reverse transcription-polymerase chain reaction
TGFβ: transforming growth factor beta
