# Supplementary material for: High mobility group protein A2 overexpression indicates poor prognosis for cancer patients: a meta-analysis
Source: Oncotarget. 2017 Dec 10;9(1):1237–47. doi: 10.18632/oncotarget.23085 (PMC5787434; doi:10.18632/oncotarget.23085)
Supplement: Supplementary file 1 [file oncotarget-09-1237-s001.pdf]

High mobility group protein A2 overexpression indicates poor prognosis for cancer patients: a meta-analysis

SUPPLEMENTARY MATERIALS

Supplementary Table 1: Characteristics of eligible studies included in the meta-analysis. See Supplementary_Table_1

Supplementary Table 2: PRISMA 2009 checklist. See Supplementary_Table_2
