# Supplementary material for: High mobility group protein A2 overexpression indicates poor prognosis for cancer patients: a meta-analysis
Source: Oncotarget. 2017 Dec 10;9(1):1237–47. doi: 10.18632/oncotarget.23085 (PMC5787434; doi:10.18632/oncotarget.23085)
Supplement: Supplementary file 2 [file oncotarget-09-1237-s002.docx]

**Supplementary Table 1.** Characteristics of eligible studies included in the meta-analysis

| **Author** | **Year** | **Country** | **Sample**  **size** | **Cancer type** | **Detection method** | **Cutoff value** | **HMGA2**  **(positive/ negative)** | **Follow-up time (month) Median (Range)** | **Outcomes** | **HR (95% CI)** | **Method of HR estimation** | **Survival Analysis** | **Quality stars (NOS)** |
| --- | --- | --- | --- | --- | --- | --- | --- | --- | --- | --- | --- | --- | --- |
| Motoyama | 2008 | Japan | 110 | GC | qRT-PCR | mRNA level (15.8±40.7) | 55/55 | 31.2(1.2-134.4) | OS | 2.00(1.32-3.15) | R | M; U | 9 |
| Kong | 2014 | China | 158 | GC | IHC | Score = 1-3 | 68/90 | 60(all) | OS | 0.98(0.34-2.33) | R | M; U | 8 |
| Wu-1 | 2016 | China | 273 | BC(a) | IHC | Score > 3 | 135/138 | NR | OS | 1.84(1.02-3.33) | R | M; U | 7 |
| Wu-2 | 2016 | China | 310 | BC(a) | IHC | Score > 3 | 202/108 | NR | OS | 2.06(1.21-3.49) | R | M; U | 7 |
| Wu | 2012 | China | 107 | HCC | IHC | Positive cells ≥ 10 % | 51/56 | 4-48（range) | OS | 1.98(1.17-3.34) | R | M; U | 7 |
| Miyazawa | 2004 | Japan | 42 | OSCC | IHC | Not reported | 31/11 | 107 (43–171) | DFS | 3.48(1.40-8.69) | R | M | 6 |
| Jun | 2015 | Korea | 110 | GC | IHC | Score = 5-8 | 72/38 | NR | RFS | 3.20(1.51–6.79) | R | M | 8 |
| Xia | 2014 | China | 124 | NPC | IHC | Score ≥ 6 | 59/65 | 50.5(5-93) | OS | 2.68(1.19-6.08) | R | M; U | 7 |
| Califano | 2014 | Italy | 113 | OC | IHC | Positive cells ≥ 10 % | 44/69 | 53.6 (median) | OS | 0.83 (0.38-1.82) | R | U | 6 |
| Na | 2016 | China | 162 | ccRCC | IHC | Score ≥ 107-290 | 76/86 | NR | OS | 3.12(1.64-5.91) | R | M; U | 6 |
| Liu | 2015 | China | 116 | NPC | IHC | Score ≥ 4 | 61/55 | 15-96 (range) | OS | 1.72(1.02-2.91) | R | M; U | 9 |
| Lee | 2014 | USA | 53 | IHCC | IHC | Positive cells ≥ 5 % | 17/36 | NR | OS | 2.20 (1.12-4.33) | R | M | 6 |
| Kim | 2014 | Korea | 74 | OC | IHC | Nuclear staining cells ≥ 50% | 30/44 | 40.1 (2-139) | OS | 1.83(0.7-4.75) | DE | U | 6 |
| Wu | 2013 | China | 51 | GB | IHC | Positive  staining cells  ≥ 50% | 31/20 | 18.6 (5-36) | OS | 3.35 (1.25-9.02) | R | M; U | 6 |
| Lee | 2015 | Korea | 170 | GC | IHC | Score ≥ 9 | 39/131 | 0-152 (range) | OS | 1.77(1.1-2.87) | DE | U | 6 |
| Wei | 2016 | China | 96 | ESCC | IHC | Score ≥ 4 | 67/29 | 30 (3-96) | OS | 1.83(1.07-3.12) | R | U | 6 |
| Günther | 2016 | Germany | 202 | HNSCC | IHC | Not reported | 54/148 | NR | OS | 1.18 (0.77-1.80) | R | M; U | 6 |
| Kwon | 2010 | Korea | 334 | HCC | RT-PCR | Histoscore | 130/204 | 51.2 (2.7–95.6) | OS | 1.74 (1.17–2.58) | R | M; U | 8 |
| Zhao | 2016 | China | 60 | TC | qRT-PCR | Not reported | 37/23 | NR | DFS | 5.79(1.07-31.39) | R | M; U | 8 |
| Wang-1 | 2011 | China | 89 | CRC | IHC | Nuclear staining cells ≥ 5% | 31/58 | NR | OS | 2.38 (1.30-4.34) | R | M | 7 |
| Wang-2 | 2011 | USA | 191 | CRC | IHC | Nuclear staining cells ≥ 5% | 70/121 | NR | OS | 2.14 (1.21-3.79) | R | M | 7 |
| Yang | 2011 | China | 148 | BC (b) | IHC | Score ≥ 2 | 77/71 | 36 (2-95) | RFS | 3.83(2.19–6.71) | R | M | 8 |
| Yu | 2016 | China | 75 | CRC | IHC | Score ≥ 4 | 39/36 | NR | OS | 1.48(0.73–3.03) | R | M; U | 7 |
| Zou | 2012 | China | 108 | GBAC | IHC | Not reported | 64/44 | NR | OS | 3.02(1.58-5.78) | R | M | 6 |
| Raskin | 2013 | USA | 330 | melanoma | IHC | Not reported | NR | NR | OS | 1.72(1.09-2.73) | R | M | 6 |
| Liu | 2016 | China | 192 | CRC | IHC | Score ≥ 4 | 117/75 | 57.6 (median) | OS | 1.12(0.57-2.20) | R | M | 8 |
| Mito | 2016 | USA | 91 | EAC | IHC | Not reported | 25/66 | NR | OS | 1.80(0.92-3.60) | R | M | 6 |
| Eide | 2016 | Norway | 156 | NSCLC | qRT-PCR | Not reported | 86/70 | 60 (all) | OS | 1.18(0.66-2.11) | R | U | 6 |
| Langelotz | 2003 | Germany | 69 | BC (a) | RT-PCR | Not reported | 21/48 | 24.7 (median) | OS | 6.38(2.22-18.34) | R | M | 6 |

GC: gastric cancer; BC (a): breast cancer; HCC: hepatocellular carcinoma; OSCC: Oral squamous cell carcinoma; NPC: nasopharyngeal carcinoma; OC: ovarian cancer; ccRCC: clear cell renal cell carcinoma; IHCC: intrahepatic cholangiocarcinoma; GB: glioblastoma; ESCC: esophageal squamous cell carcinoma; HNSCC: head and neck squamous cell carcinoma; TC: tongue cancer; CRC: colorectal cancer; BC (b): bladder carcinoma; GBAC: gallbladder adenocarcinoma; EAC: esophageal adenocarcinoma; NSCLC: non-small-cell lung cancer; NR: not reported; IHC: immunohistochemistry; qRT-PCR: quantitative real-time polymerase chain reaction; RT-PCR: reverse transcription-polymerase chain reaction; DFS: disease-free survival; OS: overall survival; RFS: recurrence-free survival; HR: hazard ratio; R: reported in text; DE: data extrapolated; U: univariate model: M: multivariate model.
